# Factors influencing husbands’ participation in antenatal care: A study in Maluku, Indonesia

**DOI:** 10.4102/hsag.v31i0.3354

**Published:** 2026-04-09

**Authors:** Magdalena Paunno, Marthin J. Maspaitella, Juliana A. Tuasela, Clara Titarsole, Bellytra Talarima, Ivy V. Lawalata, Novalin N. Titarsole, Natalia Manuhutu

**Affiliations:** 1 Department of Public Health, Faculty of Health, Universitas Kristen Indonesia Maluku, Ambon, Indonesia; 2 Department of Social and Political Affairs, Faculty of Social and Political Sciences, Universitas Kristen Indonesia Maluku, Ambon, Indonesia; 3 Department of Doctor of Theology, Faculty of Theology, Universitas Kristen Indonesia Maluku, Ambon, Indonesia; 4 Department of Medical Education, Faculty of Medicine, Universitas Pattimura, Ambon, Indonesia; 5 Department of Epidemiology, Faculty of Public Health, Universitas Kristen Indonesia Maluku, Ambon, Indonesia; 6 Department of Biotechnology, Faculty of Mathematics and Natural Sciences, Universitas Pattimura, Ambon, Indonesia; 7 Department of English Education, Faculty of Teacher Training and Education, Universitas Masamus, Merauke, Indonesia

**Keywords:** access, culture, religion, parental figures, husbands’ involvement

## Abstract

**Background:**

Husbands’ involvement in supporting standardised antenatal care (ANC) is essential for improving maternal and neonatal outcomes in geographically remote and socio-culturally complex settings. In Maluku, Indonesia, such involvement is shaped by limited-service access and entrenched cultural, religious and family power structures; yet their combined influence across pregnancy remains underexplored.

**Aim:**

This study examined how service access, cultural and religious norms, family dynamics and knowledge influence husbands’ involvement in supporting standardised ANC in Maluku province, Indonesia.

**Setting:**

The study was conducted in six remote primary health centres in Maluku province, Indonesia.

**Methods:**

A sequential explanatory mixed-methods design was applied. Quantitative data were analysed using chi-square tests and multivariable logistic regression, while qualitative data from interviews and focus group discussions were thematically analysed. Data were collected from January 2024 to June 2024.

**Results:**

Only 27% of husbands actively supported standardised ANC. Supportive roles (adjusted odds ration [AOR] = 25.2; *p* = 0.039), positive family dynamics (AOR = 2.7; *p* = 0.005) and higher ANC knowledge (AOR = 10.7; *p* = 0.024) were independently associated with involvement. Qualitative findings showed predominantly passive participation shaped by culturally sanctioned authority, religious legitimacy and community influence.

**Conclusion:**

Husbands’ limited involvement reflects gaps in knowledge, as well as the influence of family decision-making structures and socio-cultural norms. Integrating local wisdom (Bakele) into a culturally grounded, trimester-based antenatal counselling model may enhance participation.

**Contribution:**

This study informs culturally adapted ANC interventions in remote and socio-culturally complex settings.

## Introduction

Husbands’ involvement in pregnancy care (male involvement) has been recognised as an important factor in improving maternal and infant health (Falade-Fatila & Adebayo 2020). Male participation has been shown to enhance access to antenatal services, improve adherence to appointments and provide both emotional and practical support for pregnant women (Suandi, Williams & Bhattacharya 2020). Research conducted in Indonesia indicates that interventions involving husbands are associated with improved birth outcomes, particularly in low- and middle-income settings (Syamsul, Tenriola & Suriyani 2024). However, studies in Indonesia show that such involvement continues to face barriers related to institutional constraints, cultural norms and social constructions of gender that assign reproductive responsibilities primarily to women (Purnamasari 2025). Consequently, the husband’s role is often perceived merely as that of an economic provider, rather than an active partner in the pregnancy process (Eddy & Fife 2021).

In Indonesia, the government has made efforts to increase family participation in pregnancy care through the Healthy Indonesia Programme with a Family Approach (PIS-PK), which emphasises the importance of family support, including that of husbands in antenatal care (ANC) services (Morgan et al. 2022; Rakhmawati, Suryawati & Shaluhiyah 2021). Programmes such as maternal classes and prenatal couple education have been proven effective in strengthening the support provided by husbands and improving family health behaviours (Abbaspoor et al. 2023). These efforts are in line with the WHO and UNFPA recommendations that encourage male involvement in maternal and neonatal health systems to reinforce family partnerships (Gopal et al. 2020).

Nevertheless, the level of involvement of husbands in Indonesia still varies across regions (Fathanah & Pricyla 2024). Factors such as education, occupation, culture and kinship systems influence husbands’ participation in supporting antenatal check-ups (Saha 2023). In some cultures, including those in Maluku, the patrilineal social system positions men as the primary decision-makers within the household (Sopamena 2019). Decisions related to reproductive health are often made by husbands or extended family members, thereby limiting women’s autonomy and weakening partnership in pregnancy care (Tadele, Tesfay & Kebede 2019).

Interestingly, the local cultural values of Maluku actually embody a philosophy that can strengthen the husband’s role (Manuputty, Afdhal & Makaruku 2024). The concept of maintaining the ‘kintal’ or family environment to remain ‘moi’ [good or harmonious] during pregnancy positions the husband as the primary agent in caring for the wife’s physical and emotional health (Paunno et al. 2024). These traditions and beliefs, when integrated with culturally based health approaches, can serve as cultural enablers to enhance male participation in maternal health services (Melly, Magdalena & Kurniawati 2023).

However, most previous studies have primarily focused on women’s perspectives and have not extensively examined how cultural values, religion and social structures in Maluku shape the husband’s role during pregnancy (Hateyong et al. 2024). Therefore, this study aims to analyse the influence of service access, culture, religion and parental figures on husbands’ involvement in ANC (Omayma Fathy Mohammad et al. 2022). Using a mixed-methods sequential explanatory approach, this study examines both quantitative associations and the underlying social meanings of husbands’ involvement behaviours (Ghelichkhani et al. 2024).

### Aim

Conceptually, this study aims to expand the understanding of how structural determinants interact with local cultural values in family health behaviours. The findings are expected to enhance maternal health policies and programmes that focus on husband–wife partnerships, drawing on the Maluku local wisdom of ‘Bakele’ (togetherness), which highlights solidarity, collective responsibility and shared well-being during pregnancy.

## Research methods and design

### Study design

This study adopted a sequential explanatory mixed-methods design consisting of two consecutive and complementary phases. The first phase involved a quantitative approach to identify factors associated with husbands’ involvement in ANC. Findings from the quantitative phase informed the second, qualitative phase, which aimed to provide deeper explanations of the observed associations by exploring the social and cultural contexts influencing husbands’ involvement in their wives’ ANC. This design enabled the integration of measurable associations with contextual interpretations. Mixed-methods integration was achieved through joint displays, where qualitative findings were used to explain and expand upon significant quantitative results.

### Population and sampling

The population comprised husbands of pregnant women in the catchment areas of six community health centres across West Seram, South Buru and Southwest Maluku regencies (representing urban, remote, island and indigenous settings with varying ANC quality). The quantitative sample size of 300 was calculated using a single population proportion formula (assuming 30% expected involvement based on regional studies, 95% confidence interval (CI), 5% margin of error, design effect 1.5 for clustering), yielding adequate power. Convenience cluster sampling was applied based on the health centre catchment areas. Inclusion criteria included husbands living with pregnant wives and with prior pregnancy experience. Those who did not meet these criteria and unwilling to participate were excluded.

The qualitative phase included 114 informants (purposively selected for diversity and information richness): health officials (*n* = 10), health centre leaders (*n* = 8), village or sub-district heads (*n* = 12), traditional leaders (*n* = 15), religious leaders (*n* = 12), midwives (*n* = 10), traditional birth attendants (*n* = 8), health cadres (*n* = 10), pregnant women (*n* = 10), husbands (*n* = 15) and senior family figures/parents-in-law (*n* = 14). The sample size was determined by thematic saturation; such a large sample ensured comprehensive coverage of stakeholder perspectives in a culturally diverse setting.

### Data collection

#### Quantitative data

Quantitative data were collected using a structured questionnaire that was developed and adapted from previously validated instruments on male involvement in maternal health, including items used in prior Indonesian and Southeast Asian studies. The questionnaire was contextually adapted to the Maluku setting through expert review and pilot testing. It measured the following domains: (1) socio-demographic characteristics (age, education, occupation and place of residence); (2) access to antenatal counselling services and health facilities, operationalised by distance to the nearest facility (< 5 km/≥ 5 km), availability of transportation (yes/no) and frequency of counselling attendance; (3) perceived service quality, assessed using a 5-point Likert scale covering timeliness, privacy and respectful care; (4) husbands’ knowledge of ANC, measured using a composite score of items on ANC benefits, recommended timing and pregnancy risks and dichotomised at the median; (5) family dynamics, assessed through items on communication and decision-making roles within the household and dichotomised based on median scores; (6) cultural values, measured using Likert-scale items reflecting agreement with local norms and wisdom regarding husbands’ roles as protectors and clan or family heads and (7) husbands’ involvement in ANC, defined as accompaniment to at least four ANC visits combined with emotional and practical support, and categorised as a binary outcome.

The questionnaire was self-developed based on these validated sources and underwent content validation by maternal health and socio-cultural experts, yielding a content validity index greater than 0.8. Internal consistency was assessed through pilot testing, with Cronbach’s alpha of 0.82 for multi-item scales. All variables were operationalised using predefined definitions and cut-off points. Quantitative data were collected between January 2024 and June 2024 by trained enumerators under close supervision of the research team.

#### Qualitative data

Qualitative data were collected through in-depth interviews (IDIs; *n* = 74) and focus group discussions (FGDs; *n* = 8 groups, each comprising 5–8 participants). Participants were purposively selected to represent diverse stakeholder groups, including husbands, pregnant women, midwives, health cadres, religious leaders, traditional leaders, community leaders and senior family members. Semi-structured interview guides were used to explore perceptions, experiences and socio-cultural meanings related to husbands’ involvement in ANC.

Examples of guiding questions included: ‘How do cultural traditions in your community define the husband’s role during pregnancy?’; ‘What factors influence your decision to accompany your wife to antenatal care visits?’ and ‘How do religious teachings shape husbands’ involvement in pregnancy care?’ Interviews and FGDs were conducted by trained qualitative researchers fluent in local languages, lasted approximately 60 min – 90 min, were audio-recorded with participants’ consent, transcribed verbatim and translated into Indonesian for analysis.

Findings from the quantitative phase informed the qualitative inquiry by guiding the selection of probes and themes explored in greater depth, particularly for variables that showed significant associations in the regression analysis (e.g. family dynamics, husbands’ knowledge and cultural norms).

### Data analysis

#### Quantitative analysis

Quantitative data were analysed using Quantitative data were analysed using the Statistical Package for the Social Sciences (SPSS) version 26.0 (IBM Corp., Armonk, NY, United States). The dependent variable was husbands’ involvement in standardised ANC, operationalised as a binary outcome (active vs. passive involvement). Active involvement was defined as husbands accompanying their wives to at least four recommended ANC visits and providing emotional or practical support during pregnancy, while passive involvement referred to limited or no participation.

Independent variables included socio-demographic characteristics (age, education level and occupation); access to health services (distance to health facility, transportation availability and perceived affordability of services); husbands’ knowledge of ANC (composite score derived from items assessing knowledge of ANC benefits, recommended timing and pregnancy risks, dichotomised at the median); family decision-making dynamics (measured through items on household communication and authority, dichotomised at the median); cultural norms (agreement with local values regarding husbands’ roles as protectors and family or clan leaders) and religious values (perceived religious expectations related to husbands’ responsibilities during pregnancy).

Bivariate analysis was conducted using chi-square tests to assess associations between independent variables and husbands’ involvement in ANC at a significance level of *p* < 0.05 with 95% confidence intervals. Variables that demonstrated significant associations in the bivariate analysis (*p* < 0.05), as well as variables considered theoretically important based on previous literature, were entered into a multivariable binomial logistic regression model to estimate crude odds ratios (COR) and adjusted odds ratios (AOR) while controlling for potential confounders.

#### Qualitative analysis

Qualitative data were analysed using thematic analysis following the six-phase framework proposed by Braun and Clarke, comprising data familiarisation, initial coding, searching for themes, reviewing themes, defining and naming themes and interpretation. Coding was conducted iteratively and inductively to identify recurring patterns related to husbands’ involvement in ANC. This process resulted in five overarching themes: (1) husbands’ participation in pregnancy care, (2) social and cultural power structures within families, (3) spiritual and religious values shaping involvement, (4) access to information and health communication and (5) the role of culturally contextualised educational media.

#### Rigour, validity and reliability

For the quantitative component, internal consistency of multi-item scales was assessed using Cronbach’s alpha, with values exceeding 0.70 indicating acceptable reliability. Content validity was established through expert review involving maternal health specialists and socio-cultural experts to ensure relevance, clarity and contextual appropriateness of questionnaire items.

For the qualitative component, trustworthiness was ensured through multiple strategies, including triangulation of data sources (across participant groups and data collection methods), peer debriefing within the research team to refine coding and theme development and member checking with selected informants to verify the accuracy and credibility of interpretations. These procedures enhanced the credibility, dependability, confirmability and transferability of the qualitative findings.

### Ethical considerations

This study received ethical approval from the appropriate institutional ethics review board (Fakultas Kesehatan Masyarakat, Universitas Airlangga, dengan nomor sertifikat 229/EA/KEPK/2025). All participants provided written informed consent after receiving a clear explanation of the study’s objectives, procedures, potential risks and benefits. Participation was voluntary, and participants were informed of their right to refuse or withdraw from the study at any stage without any consequences. Confidentiality and anonymity were strictly maintained by removing personal identifiers and securely storing all data, which were accessible only to the research team. The study was conducted in accordance with internationally recognised ethical principles for research involving human participants.

## Results

### Quantitative results

A total of 300 husbands participated in the study, of whom only 27% were actively involved in standardised ANC, defined as accompanying their wives to ANC visits and providing emotional and practical support. As shown in Table 1, bivariate analysis demonstrated that husbands’ involvement in standardised ANC was significantly associated with several socio-demographic, access-related and socio-cultural factors. Older husbands (≥ 20 years) were more likely to be involved than younger husbands (*p* = 0.028), and higher educational attainment was significantly associated with active involvement (*p* = 0.016). Occupational status was also relevant, with entrepreneurs showing higher participation compared to husbands employed in the private sector (*p* = 0.010).

**TABLE 1:** Bivariate analysis of factors associated with husbands’ involvement in standardised antenatal care in Maluku province, Indonesia.

Variable	Husband’s involvement				*X* ^2^	*p*-value
	Yes		No			
	*n*	%	*n*	%		
**Husband’s age (years)**	-	-	-	-	-	0.028*
< 20	26	36.6	45	63.4	4.367	-
≥ 20	55	24.0	174	76.0	-	-
**Husband’s education**	-	-	-	-	-	0.016*
Higher (≥ Senior high school)	41	21.9	146	78.1	6.487	-
Lower (< Senior high school)	40	35.4	73	64.6	-	-
**Husband’s occupation**	-	-	-	-	-	0.010*
Entrepreneur	42	35.6	76	64.4	7.287	-
Private sector	39	21.4	143	78.6	-	-
**Access to ANC counselling services**	-	-	-	-	-	< 0.001***
Yes	40	41.7	56	58.3	15.408	-
No	41	20.1	163	79.9	-	-
**Access to health facilities**	-	-	-	-	-	< 0.001***
Easy	36	41.9	50	58.1	13.508	-
Difficult	45	21.0	169	79.0	-	-
**ANC facilities and counselling**	-	-	-	-	-	< 0.001***
Sufficient	40	41.2	57	58.8	14.742	-
Insufficient	41	20.2	162	79.8	-	-
**Husband’s support during pregnancy**	-	-	-	-	-	< 0.001***
Yes	42	44.2	53	55.8	20.893	-
No	39	19.0	166	81.0	-	-
**Cultural values and local wisdom**	-	-	-	-	-	0.005**
Yes	38	37.6	63	62.4	8.719	
No	43	21.6	156	78.4	-	-
**Social stigma**	-	-	-	-	-	0.203
Positive	27	32.9	55	67.1	2.011	-
Negative	54	24.8	164	75.2	-	-
**Family dynamics (the role of parental figures)**	-	-	-	-	-	< 0.001***
Joint participation	42	41.6	59	58.4	16.431	-
No joint participation	39	19.6	160	80.4	-	-
**Husband’s knowledge of ANC and family role**	-	-	-	-		< 0.001***
Good	43	44.8	53	55.2	22.673	-
Poor	38	18.6	166	81.4	-	-

ANC, antenatal care.

* , *p* < 0.05;

** , *p* < 0.01;

*** , *p* < 0.001.

Access-related variables exhibited the strongest associations with husbands’ involvement. Husbands who reported access to ANC counselling services, ease of reaching health facilities and sufficient ANC facilities and counselling quality were significantly more likely to be actively involved (all *p* < 0.001). These findings highlight the central role of service availability and their perceived quality in facilitating husbands’ participation in their wives’ ANC.

Socio-cultural and family-related factors were also significantly associated with involvement. Husbands who provided active support during pregnancy, endorsed cultural values and local wisdom regarding husbands’ roles and participated in joint family decision-making involving parental figures demonstrated substantially higher involvement in standardised ANC (all *p* < 0.01). Notably, husbands’ knowledge of ANC and their perceived family leadership role showed the strongest bivariate association with involvement (χ^2^ = 22.673, *p* < 0.001).

In contrast, perceived social stigma was not significantly associated with husbands’ involvement in standardised ANC (*p* = 0.203), suggesting that normative social pressures alone may be less influential than structural access and family decision-making dynamics in this setting.

Multivariable logistic regression analysis demonstrated adequate model fit (Hosmer–Lemeshow goodness-of-fit test, *p* = 0.412) and moderate explanatory power (Nagelkerke *R*^2^ = 0.48). As presented in Table 2, three factors remained independently associated with husbands’ active involvement in standardised ANC after adjustment for potential confounders.

**TABLE 2:** Multivariable binomial logistic regression model of factors associated with husbands’ involvement in standardised antenatal care in Maluku province, Indonesia.

Variable	Crude odds ratio	95% CI	*p*-value	Adjusted odds ratio	95% CI	*p*-value
**Husband’s age (years)**	1.8	1.0–3.2	-	0.4	0.1–1.1	0.088
< 20	-	-	< 0.038	-	-	-
≥ 20	-	-	-	-	-	-
**Husband’s education**	0.5	0.3–0.9	-	3.7	0.7–18.9	0.110
Higher	-	-	< 0.011	-	-	-
Lower	-	-	-	-	-	-
**Husband’s occupation**	2.0	1.2–3.4	-	0.7	0.1–5.3	0.764
Entrepreneur	-	-	< 0.007	-	-	-
Private sector	-	-	-	-	-	-
**Access to ANC counselling services**	2.8	1.7–4.8	-	0.0	0.0–1.2	0.62
Yes	-	-	< 0.000	-	-	-
No	-	-	-	-	-	-
**Access to health facilities**	2.7	1.6–4.6	-	1.7	0.5–6.0	0.443
Easy	-	-	< 0.000	-	-	-
Difficult	-	-	-	-	-	-
**ANC facilities and counselling**	2.8	1.6–4.7	-	1.1	0.3–4.1	0.903
Sufficient	-	-	< 0.000	-	-	-
Insufficient	-	-	-	-	-	-
**The role and support of the husband**	3.4	2.0–5.8	-	25.2	1.2–536.9	0.039*
Yes	-	-	< 0.000	-	-	-
No	-	-	-	-	-	-
**The role of cultural values and local wisdom**	2.2	1.3–3.7	-	0.9	0.3–2.7	0.855
Yes	-	-	< 0.003	-	-	-
No	-	-	-	-	-	-
**Family dynamics (the role of parental figures)**	2.9	1.7–5.0	-	2.7	1.3–5.3	0.005**
Joint participation	-	-	< 0.000	-	-	-
No joint participation	-	-	-	-	-	-
**Husband’s knowledge about ANC and role as family head**	3.5	2.1–6.1	-	10.7	1.4–82.7	0.024*
Good	-	-	< 0.000	-	-	-
Poor	-	-	-	-	-	-

ANC, antenatal care; CI, confidence interval.

* , *p* < 0.05;

** , *p* < 0.01.

Husbands who provided active support during pregnancy – such as accompanying their wives to ANC visits, offering encouragement and actively seeking pregnancy-related information – were substantially more likely to be involved in standardised ANC compared with those who did not provide such support (AOR = 25.2; 95% CI: 1.2–536.9; *p* = 0.039). Supportive family dynamics characterised by joint decision-making involving parental figures were also significantly associated with higher involvement (AOR = 2.7; 95% CI: 1.3–5.3; *p* = 0.005). In addition, husbands with good knowledge of ANC and a clear understanding of their role as family head demonstrated significantly greater participation in standardised ANC (AOR = 10.7; 95% CI: 1.4–82.7; *p* = 0.024).

In contrast, socio-demographic characteristics (age, education and occupation) and structural access-related factors – including access to ANC counselling services, ease of reaching health facilities and perceived adequacy of ANC facilities and counselling quality – did not remain statistically significant in the adjusted model. These findings suggest that, in this setting, husbands’ involvement in standardised ANC is primarily driven by socio-cultural and knowledge-related factors rather than structural access alone.

### Qualitative results

Qualitative thematic analysis (see Table 3) resulted in five major themes that explain the socio-cultural context of husbands’ involvement in their wives’ ANC: (1) husbands’ participation is still passive, (2) social structures and family power dynamics influence decision-making, (3) religious values provide moral legitimacy for the husband’s role, (4) community leaders are effective agents of change in fostering awareness and male role modelling and (5) husbands need educational media based on local languages and trimester-based guides to strengthen sustained involvement.

**TABLE 3:** Thematic analysis matrix of qualitative research.

Theme/sub-theme	Category	Supporting informant quotes
Theme 1: Limited physical involvement of husbands	Traditional gender perceptions	‘The husband just sees his wife as healthy, doing all the work, usually just accompanying her to the midwife.’ (Midwife)
Theme 1: Husband’s participation Social barriers to emotional support	Social norms and embarrassment	‘When the husband is present, the wife feels more at ease. But many are too embarrassed to join.’ (Pregnant mother)
Theme 2: Socio-cultural barriers Extended family dominance	Decision making by parents and in-laws	‘Decisions are usually made by the parents or in-laws.’ (Senior family figure)
Theme 2: Socio-cultural barriers Customary norm practice gap	Cultural ambivalence	‘In custom, the husband is obliged to take care of the wife, but in practice it is left to the family.’ (Customary leader)
Theme 3: Role of religion Moral reinforcement	Religious authority	‘A husband is the leader of the family. If it’s delivered through a sermon, it’s more likely to be heard.’ (Religious leader)
Theme 4: Agents of change Social authority influence	Customary and village leaders	‘If the customary leader speaks, the husband is more likely to comply.’ (Village head)
Theme 5: Access to information Community-based media	Simple local language	‘If there’s a video using simpler language, it would be easier to understand, not just health terms.’ (Husband)
Theme 5: Access to information Pregnancy stage guidance	Trimester-specific information	‘If there were guidelines for each trimester, the husband would know what to do.’ (Parent)

## Discussion

### Interpretation of findings

The research findings reveal that husbands’ involvement in pregnancy care is shaped by an interplay of social, cultural and knowledge-related factors (Boniphace et al. 2021). Quantitatively, husbands’ active support role strongly predicts involvement in ANC accompaniment (AOR = 25.2; 95% CI: 1.2–536.9; *p* = 0.039), with emotional and informational support defined as encouragement, shared decision making and information-seeking increasing participation up to 25-fold. Supportive family dynamics, including joint decision-making with parental figures, were significantly associated with higher levels of husbands’ involvement in standardised ANC (AOR = 2.7; 95% CI: 1.3–5.3; *p* = 0.005). This finding is consistent with previous studies highlighting the reinforcing roles of wives and parental figures in promoting ANC practices (Wood et al. 2024). In addition, husbands’ knowledge about ANC and his awareness as the head of the family are significantly associated with the likelihood of them accompanying their wives for ANC visits (AOR = 10.7; 95% CI: 1.4–82.7; *p* = 0.024), indicating that understanding the benefits of ANC strengthens decision-making ability in emergency situations.

Conversely, structural factors such as age, education, type of work, access to services and quality of facilities did not show a significant association after adjustment. These results emphasise that husbands’ involvement is more influenced by socio-cultural mechanisms and masculinity values that shape perceptions of reproductive responsibility.

Qualitative data complement these results by showing limited and discontinuous husband participation, constrained by gender norms that view pregnancy as a woman’s domain. Better-informed husbands were more likely to accompany wives to health facilities, while social barriers (e.g. embarrassment, long waiting times) hindered emotional support. Parental/in-law dominance often restricted husbands’ autonomy in reproductive decisions although supportive parental roles could motivate involvement. Community and religious leaders play key roles in conferring social legitimacy for active participation.

Religious and spiritual values provide a solid moral foundation, placing the husband as the family’s imam who cares for his wife both physically and emotionally. The involvement of religious leaders in prenatal education has been proven to increase male participation. In the context of Maluku culture, these values are manifested in the view that the husband is responsible for maintaining the harmony and safety of the pregnancy environment to keep it healthy and protected, as a form of respect for the family’s dignity. Access to information and contextual educational media is also an important factor (Mapunda et al. 2022). Respondents stated that media created using non-medical language and tailored to the stages of pregnancy are considered more effective in encouraging real behavioural changes (Oziwele & Ogerugba 2024). In addition, the involvement of community leaders also increases trust and acceptance of the health messages conveyed (Lansing et al. 2023; Petiwala et al. 2021).

Leveraging the values of ‘Bakele’ or togetherness as the basis for intervention allows for the design of ANC programmes that position husbands and extended family figures as active partners through family counselling sessions, official invitations for husbands and talks by traditional and religious leaders. This approach not only increases social legitimacy for men’s presence in health facilities but also speeds up emergency decision-making, which contributes to reducing delays in maternal and perinatal care.

These results align with Ethiopian and Zambian studies highlighting emotional support and family communication as key to men’s maternal service participation (Wood et al. 2024). Global evidence shows that religious values and community legitimacy can shift masculinity norms towards inclusivity. Integrating local cultural values strengthens intervention relevance and acceptance (Joseph & Maluka 2021; Paunno et al. 2024; Rahayu et al. 2023; Sahani et al. 2024; Shaluhiyah, Suryoputro & Indraswari 2023). The findings extend prior literature by detailing socio-cultural drivers in Maluku/eastern Indonesia (regional surveys) and resonate with studies on ANC quality in India, Pakistan and Africa, where cultural barriers limit involvement while family-centred approaches offer solutions (Atif et al. 2023; Gessesse et al. 2024; Girotra et al. 2023; Harrison et al. 2024; Nihal & Shekhar 2024).

### Integration with existing literature

These findings align with studies in Ethiopia and similar African contexts, where emotional support, family communication and knowledge significantly enhance men’s participation in maternal services (Gessesse et al. 2024; Wood et al. 2024). Emotional support serves as a key catalyst for maternal health decision-making, while open family dialogue reinforces service utilisation – patterns consistent with the strong effects of family dynamics and husbands’ knowledge observed here.

Global evidence indicates that religious values and legitimacy from community leaders can shift traditional masculinity norms towards greater participation in reproductive health (Joseph & Maluka 2021; Shaluhiyah et al. 2023). In patriarchal settings, religious endorsement provides moral justification for men’s involvement, mirroring the qualitative emphasis on spiritual legitimacy and leader roles in Maluku.

Integrating local cultural values such as ‘Bakele’ (togetherness) and ‘Moi Kintal’ harmony into antenatal services improves relevance, trust and acceptance, as culturally adapted interventions align health messages with community norms (Mapunda et al. 2022; Shiferaw & Minale 2025). This approach addresses gaps in prior quantitative-dominant research by detailing socio-cultural mechanisms in Maluku/eastern Indonesia, where involvement of husbands in their wives’ pregnancy care remains lower than national averages. It also resonates with evidence from India, Pakistan and broader African settings, where cultural barriers limit male engagement, but family-centred, norm-sensitive strategies improve outcomes (Atif et al. 2023; Gessesse et al. 2024; Harrison et al. 2024; Nihal & Shekhar 2024). The WHO guidelines encourage partner-inclusive psychosocial support to reduce perinatal burdens (World Health Organization 2023).

### Implications for practice

These findings offer practical guidance for community-based interventions (Shaluhiyah et al. 2023). Firstly, antenatal counselling should incorporate relevant cultural and spiritual values to strengthen the meaning and acceptance of health messages. A model grounded in local wisdom can reinforce husbands’ responsibility for family honour and well-being during pregnancy. Secondly, engaging traditional leaders, religious figures and parents in educational activities enhances social legitimacy and promotes active participation of husbands in their wives’ ANC (Joseph & Maluka 2021; Paunno et al. 2024; Rahayu et al. 2023; Sahani et al. 2024). Thirdly, developing trimester-specific educational media improves communication effectiveness and supports husbands’ evolving roles across pregnancy stages.

Family-centred interventions that involve the entire social unit beyond pregnant women and midwives can effectively reduce decision-making delays (three delays model) and improve maternal and neonatal outcomes. Embedding these results into primary care policies will foster a culturally responsive health system in settings like Maluku.

### Limitations

The sequential explanatory mixed-methods design enabled the integration of quantitative and qualitative findings, providing a richer understanding of the socio-cultural context of husbands’ involvement in ANC. However, causal inferences cannot be drawn due to the cross-sectional nature of the quantitative phase and the use of a convenience cluster sampling strategy, which was applied to capture regional diversity and local socio-cultural variation in Maluku. The complementary use of quantitative and qualitative approaches enhanced the overall robustness of the findings. Although confidence intervals were wide for some estimates, this likely reflects contextual heterogeneity and the socio-cultural complexity of the study setting, as well as variability across geographically remote and culturally diverse communities.

### Recommendations

Family-based ANC involving married couples (bakele) should be made an integral part of maternity classes to strengthen communication and joint decision-making. The development of educational media using a progressive multi-channel community communication approach from Hulur to Hilir, tailored to the stages of pregnancy, childbirth and the neonatal period, will help husbands understand their role at each phase of the child’s development.

In the future, longitudinal and quasi-experimental studies are needed to assess the long-term impact of bakele ANC interventions on maternal and neonatal outcomes, while also opening opportunities to replicate ANC programmes in Indonesia.

## Conclusion

This study emphasises that husbands’ involvement in pregnancy care is influenced by a complex interaction of institutional factors, cultural values, religion and parental figures. Community support, husbands’ knowledge about ANC and harmonious family dynamics as indicators of a supportive environment are the main determinants that strengthen husbands’ participation. Conversely, cultural norms and traditional social structures can act as both obstacles and opportunities, depending on the extent to which health interventions are able to adapt to local values and practices.

The Bakele approach shows promise as a culturally grounded model that can strengthen family and community partnerships in maternal and neonatal health. These findings provide important guidance for the development of maternal healthcare policies and practices. Integrating local cultural values into ANC programmes needs to be reinforced to ensure greater community acceptance, especially in areas with strong kinship systems. Involving social and spiritual figures such as traditional leaders, religious authorities and senior elders can enhance social legitimacy as well as the effectiveness of health messages.
